# Supplementary material for: CMTCN: a web tool for investigating cancer-specific microRNA and transcription factor co-regulatory networks
Source: PeerJ. 2018 Nov 12;6:e5951. doi: 10.7717/peerj.5951 (PMC6237116; doi:10.7717/peerj.5951)
Supplement: Table S1 — CMTCN utilized information provided by established regulatory databases of both predicted and experimentally validated interactions. [file peerj-06-5951-s001.pdf]

Interaction type	Source	Confidence	Number of interactions	Version/Release date
TF -> Gene	ITFP	Experimental	48861	Aug.2008
	TRRUST v2	Experimental	8427	V2.0
	HTRIdb	Experimental	52467	Aug.2012
	TRED	Predicted	7042	Jan.2007
miRNA -> Gene/TF	miR2Disease	Experimental	603	Apr.2008
	miRTarBase	Experimental	380639	V7.0
	miRecords	Experimental	1715	Apr.2013
	starBase	Predicted	419678	V1.0
	TargetScan	Predicted	100031	V7.0
TF -> miRNA	mirTrans	Experimental	201364	Oct.2017
	PuTmiR	Predicted	10147	V1.1
