# Supplementary material for: CMTCN: a web tool for investigating cancer-specific microRNA and transcription factor co-regulatory networks
Source: PeerJ. 2018 Nov 12;6:e5951. doi: 10.7717/peerj.5951 (PMC6237116; doi:10.7717/peerj.5951)
Supplement: Table S2 — CMTCN curated cancer-related genes/miRNAs manually for 33 types of cancer by referring to cancer gene/miRNA databases. [file peerj-06-5951-s002.pdf]

Source / Cancer	IntOGen	SEGreg	TissGDB
ACC	0	265	10
BLCA	150	88	98
BRCA	175	85	38
CESC	0	119	105
CHOL	0	106	0
COAD	92	154	115
DLBC	9	499	0
ESCA	96	82	55
GBM	73	429	186
HNSC	160	253	0
KICH	0	428	262
KIRC	97	262	262
KIRP	0	203	262
LAML	32	1326	164
LGG	47	794	186
LIHC	29	577	376
LUAD	176	88	102
LUSC	142	70	102
MESO	0	147	0
OV	79	137	127
PAAD	20	159	134
PCPG	0	602	10
PRAD	85	242	112
READ	92	187	0
SARC	0	147	50
SKCM	242	139	212
STAD	170	76	138
TGCT	0	449	1272
THCA	0	216	72

THYM	0	261	34
UCEC	147	136	43
UCS	0	141	43
UVM	0	403	109
