# Supplementary material for: CMTCN: a web tool for investigating cancer-specific microRNA and transcription factor co-regulatory networks
Source: PeerJ. 2018 Nov 12;6:e5951. doi: 10.7717/peerj.5951 (PMC6237116; doi:10.7717/peerj.5951)
Supplement: Table S3 — CMTCN curated cancer-related genes/miRNAs manually for 33 types of cancer by referring to cancer gene/miRNA databases. [file peerj-06-5951-s003.pdf]

Cancer \ Source	HMDD2.0	miRCancer	miR2Disease	PhenomiR
ACC	54	6	0	0
BLCA	100	50	0	31
BRCA	206	140	5	276
CESC	53	57	2	86
CHOL	13	11	0	0
COAD	85	124	0	0
DLBC	8	0	1	0
ESCA	74	96	0	1
GBM	101	57	0	115
HNSC	75	28	4	202
KICH	116	10	1	0
KIRC	7	19	1	4
KIRP	0	43	1	0
LAML	68	20	3	149
LGG	76	103	0	0
LIHC	219	200	0	115
LUAD	135	90	8	239
LUSC	97	117	3	0
MESO	32	0	0	0
OV	116	70	5	189
PAAD	104	78	2	200
PCPG	12	0	0	0
PRAD	123	87	4	202
READ	168	49	4	159
SARC	55	91	0	0
SKCM	3	0	0	0
STAD	175	152	0	140
TGCT	7	0	0	0
THCA	51	34	0	12

THYM	15	0	0	0
UCEC	104	31	0	0
UCS	2	0	0	0
UVM	0	53	1	225
