# Supplementary material for: CMTCN: a web tool for investigating cancer-specific microRNA and transcription factor co-regulatory networks
Source: PeerJ. 2018 Nov 12;6:e5951. doi: 10.7717/peerj.5951 (PMC6237116; doi:10.7717/peerj.5951)
Supplement: Table S4 [file peerj-06-5951-s004.pdf]

Nodes	Score	Group
MELK	1	Gene
CLU	0.67787	Gene
PIGR	0.49585	Gene
SNX5	0.41835	Gene
DAPK2	0.37870	Gene
ITGB3	0.36933	Gene
EPHA4	0.30666	Gene
CALM1	0.27667	Gene
SNX22	0.26060	Gene
TG	0.22607	Gene
