# Supplementary material for: CMTCN: a web tool for investigating cancer-specific microRNA and transcription factor co-regulatory networks
Source: PeerJ. 2018 Nov 12;6:e5951. doi: 10.7717/peerj.5951 (PMC6237116; doi:10.7717/peerj.5951)
Supplement: Table S5 — With KEGG pathway enrichment, CMTCN found 11 significant pathways, all of which were related to cance. [file peerj-06-5951-s005.pdf]

Description	Gene ratio	pvalue	p.adjust	qvalue	Count	Gene symbol
Pathways in cancer	21/66	0	0	0	21	E2F1/PAX8/SPI1/FOS/STAT1/NFKB1/AR/RELA/PPARG/ETS1/LEF1MITF/PPARD/MYC/HIF1A/DAPK2/FN1/VEGFC/EGFR/PTEN/VEGFA
Cell cycle	11/66	0.00002	1E-05	0	11	MCM4/E2F4/MCM5/E2F1/MCM2/MCM3/MCM6/MCM7/TFDP1/MYC/BUB1B
DNA replication	6/66	0	0.00013	0.0001	6	MCM4/MCM5/MCM2/MCM3/MCM6/MCM7
Bladder cancer	6/66	1E-05	0.00023	0.0002	6	E2F1/MYC/DAPK2/VEGFC/EGFR/VEGFA
Prostate cancer	8/66	1E-05	0.00023	0.0002	8	CREB1/E2F1/NFKB1/AR/RELA/LEF1/EGFR/PTEN
Pancreatic cancer	7/66	2E-05	0.00034	0.0003	7	E2F1/STAT1/NFKB1/RELA/VEG

						FC/EGFR/VEGFA
Acute myeloid leukemia	6/66	9E-05	0.00117	0.0009	6	SPI1/NFKB1/RELA/LEF1/PPARD/MYC
Thyroid cancer	4/66	0.0004	0.00475	0.0036	4	PAX8/PPARG/LEF1/MYC
Small cell lung cancer	6/66	0.0006	0.00582	0.0044	6	E2F1/NFKB1/RELA/MYC/FN1/PTEN
Endometrial cancer	4/66	0.0039	0.03458	0.0259	4	LEF1/MYC/EGFR/PTEN
TGF-β signaling	5/66	0.0042	0.03458	0.0259	5	E2F4/TFDP1/SP1/MYC/ACVR1
